# The Crucial Roles of Diet, Microbiota, and Postbiotics in Colorectal Cancer

**DOI:** 10.1007/s13668-024-00525-z

**Published:** 2024-03-14

**Authors:** Rüya Kuru-Yaşar, Özlem Üstün-Aytekin

**Affiliations:** Department of Nutrition and Dietetics, Hamidiye Faculty of Health Sciences, University of Health Sciences, 34668 Istanbul, Türkiye

**Keywords:** Diet, Microbiota, Dysbiosis, Postbiotics, Colorectal cancer

## Abstract

**Purpose of Review:**

Colorectal cancer is the second deadliest cancer in the world, and its prevalence has been increasing alarmingly in recent years. After researchers discovered the existence of dysbiosis in colorectal cancer, they considered the use of probiotics in the treatment of colorectal cancer. However, for various reasons, including the low safety profile of probiotics in susceptible and immunocompromised patient5s, and the risk of developing antibiotic resistance, researchers have shifted their focus to non-living cells, their components, and metabolites. This review aims to comprehensively evaluate the literature on the effects of diet, microbiota, and postbiotics on colorectal cancer and the future of postbiotics.

**Recent Findings:**

The link between diet, gut microbiota, and colorectal cancer has been established primarily as a relationship rather than a cause-effect relationship. The gut microbiota can convert gastrointestinal tract and dietary factors into either onco-metabolites or tumor suppressor metabolites. There is serious dysbiosis in the microbiota in colorectal cancer. Postbiotics appear to be promising agents in the prevention and treatment of colorectal cancer.

**Summary:**

It has been shown that various postbiotics can selectively induce apoptosis in CRC, inhibit cell proliferation, growth, invasion, and migration, modulate the immune system, suppress carcinogenic signaling pathways, maintain intestinal epithelial integrity, and have a synergistic effect with chemotherapy drugs. However, it is also reported that some postbiotics are ineffective and may be risky in terms of safety profile in some patients. Many issues need to be researched about postbiotics. Large-scale, randomized, double-blind clinical studies are needed.

## Introduction

Colorectal cancer (CRC), which includes cancers that occur in the colon and rectum, is the third most common type of cancer in oncologic pathology, following breast and lung cancers. In 2020, nearly 2 million cases were diagnosed. While effective screening techniques exist that could reduce the number of deaths, CRC is still the second most common cause of cancer-related death. The International Agency for Research on Cancer estimates that the global burden of CRC will increase by 56% between 2020 and 2040, with more than 3 million new cases expected annually [https://publications.iarc.fr/, Date of Access: 07.12.2023].

Most CRCs occur sporadically due to genetic mutations and epigenetic modifications in the human genome. These modifications alter the signaling pathways that regulate cell behavior, enabling the progression from normal mucosa to carcinoma [1]. About two-thirds of all CRCs are sporadic, while the remaining third are familial or inherited [2]. The most common hereditary CRC is Lynch syndrome, accounting for 2–3% of cases, followed by familial adenomatous polyposis responsible for 0.5–1% of cases, and other hereditary variants such as MUTYH-associated polyposis, Peutz-Jeghers syndrome, and others, represent less than 1% of cases [3–5]. In particular, the high turnover rate of intestinal epithelium makes this tissue a focal point for malignant transformations. However, in the first stage, CRC usually starts as a neoplastic or non-neoplastic polyp in the inner wall of the colon or rectum. The larger the polyps, the greater their malignant potential. The most common type is the adenomatous polyp, which occurs in the glandular cells that produce mucus from these polyps. Adenomas formed in the inner wall of the colorectum are histologically neoplastic. If they become malignant, they are referred to as adenocarcinomas, which make up 96% of all CRCs [6].

It is thought that the development of CRC can result from one or a combination of three different mutational pathways: chromosomal instability (CIN) characterized by an increase in chromosomal aneuploidies; epigenetic changes that cause silencing of tumor suppressor genes, caused by hypermethylation of CpG regions known as the CpG island methylator phenotype (CIMP); and microsatellite instability (MSI) caused by impairment of DNA mismatch repair. Approximately 15% of all CRC cases exhibit MSI, while CIMP is found in 10–40%, and the remaining 85% are associated with CIN. CIN is linked to the mutation of tumor suppressor genes, including adenomatous polyposis coli (APC), P53, Mothers Against Decapentaplegic homolog 2 (SMAD2), SMAD4, deleted in colorectal cancer (DCC), and oncogenes Kirsten rat sarcoma virus (K-ras) and β-catenin [1, 7, 8]

Elective surgery, radiotherapy, and chemotherapy methods are used to treat CRC [9]. Management of CRC is difficult due to late diagnosis, high recurrence rates, and multidrug resistance. Due to the problems caused by the side effects of these treatments and their negative effects on the healing process, different treatment methods are continually being developed. Despite the widespread use of chemotherapy agents in CRC, their limited effectiveness, selectivity, low bioavailability, poor solubility, and non-specific biodistribution between cancer and normal cells/tissues are significant challenges. Increasing cancer deaths and the high cost of treatment increase the interest in new therapeutic approaches and the discovery of chemo-preventive agents from natural sources [10].

Environmental factors and lifestyle, including diet, are crucial in the development of CRC [11]. The interaction between microbiota and CRC has been the focus of research lately [12]. The microbiota refers to the community of microorganisms, including bacteria, fungi, viruses, and yeasts, present in a defined environment and residing on or within human tissues such as the skin, lung, oral mucosa, and the urogenital and gastrointestinal tracts. It has been estimated that close to 95% of these microbes are in our gut, particularly the large intestine [https://internationalprobiotics.org/home/, Date of Access: 07.11.2023]. Only 1.9% of the gut microbiome is estimated to be heritable, and more than 20% of the variance in microbiome β-diversity can be inferred from environmental factors related to diet and lifestyle [12, 13]. Diet can reshape the community structure of the gut microbiota and affect its function by modulating the production of metabolites [12]. CRC is associated with a process known as dysbiosis, which includes the depletion and/or enrichment of certain microbiota members and their metabolic functions [14]. With a better recognition of the role of the microbiota in cancer pathogenesis, the potential of microbiota-based therapeutics has become an increasingly researched topic in cancer therapy. The connection between gut microbiota, dysbiosis, and CRC has led researchers to explore the effects of probiotics in CRC prevention and treatment [15]. The oral use of probiotics is widespread today; however, the safety profile of viable probiotics remains a topic of debate. Therefore, research in this direction seems to be shifting toward postbiotics [16]. Postbiotics are defined as “a preparation of inanimate microorganisms and/or their components that confer a health benefit on the host” [17••]. While postbiotics do not contain live microorganisms, they are thought to exert a beneficial health effect through similar mechanisms characteristic of probiotics while minimizing the risks associated with their intake [18]. Considering all this information, our review focuses on the effects of diet, microbiota, and the increasingly popular postbiotics in CRC.

## Diet and CRC

Comprehensive research suggests that diet may play both a causal and protective role in the development of colon cancer [19]. In 2018, the World Cancer Research Fund published a report analyzing 99 studies involving over 29 million adults and more than 247,000 cases of CRC worldwide. This report has identified the following as solid evidence of relationships between diet, nutrition, physical activity, and risk of CRC. Body fatness; consuming processed meat, red meat, and alcoholic beverages; and being tall can increase the risk of CRC. In addition, it has been stated that smoking and inflammatory bowel diseases (such as Chron’s disease and ulcerative colitis) increase the risk of CRC (dietandcancerreport.org, Date of Access: 10.12.2023). In a study involving 2502 CRC cases and 2538 controls, a positive correlation was found between the dietary inflammatory index and CRC [20]. Another study with 400 CRC patients and 400 healthy individuals identified several risk factors for CRC, including obesity, active and passive smoking, low physical activity, and high consumption of red meat and salt [6].

Chronic consumption of the Western diet has been shown to exacerbate colitis and promote colorectal tumor development in mice by activating proinflammatory and abnormal immune response pathways in the colon [21]. A prospective cohort meta-analysis, involving 80,110 cases, determined that CRC had the highest number of diet-related cases. Low consumption of dairy products and whole grains, along with high consumption of processed meat, were contributing factors [22]. In another prospective study, it has been shown that participants who consumed an average of 76 g of processed and red meat per day had a higher risk of CRC compared to those consuming 21 g per day [23].

It is known that cooking meat at high temperatures causes the formation of heterocyclic amines and polycyclic aromatic hydrocarbons, which experimental studies have linked to CRC. Additionally, the high level of heme iron in red meat has been shown to promote CRC by stimulating the endogenous formation of carcinogenic N-nitroso compounds. Processed meat also contains exogenous N-nitroso compounds, which could potentially be carcinogenic [24]. The pre-diagnosis processed meat diet model is associated with higher tumor recurrence, metastasis, and mortality in CRC patients [25]. High consumption of red meat has been shown to increase the levels of oncogenic mature miRNAs, including miR17-92 cluster miRNAs and miR21, in the human rectal mucosa, followed by the addition of butyrylated-resistant starch to the diet, with miR17-92 level returning to baseline and fecal butyrate level increasing [26].

Considering data from three large prospective cohorts, it has been stated that high consumption of ultra-processed foods is associated with the risk of CRC. Among subgroups of ultra-processed foods, higher consumption of meat/poultry/seafood-based ready-to-eat products and sugar-sweetened beverages in men, as well as ready-to-eat/heat-mixed dishes in women, is associated with an increased risk of CRC [27]. Consumption of a plant-based diet high in refined grains and sugar was associated with a high incidence of CRC [28].

According to the report of the World Cancer Research Fund, engaging in physical activity, consuming whole grains, incorporating foods with dietary fiber, consuming dairy products, and taking calcium supplements can reduce the risk of CRC at the level of solid evidence. Furthermore, using anti-inflammatory drugs for 5 years or more and hormone therapy in postmenopausal women have been found to reduce the risk of CRC (dietandcancerreport.org, Date of Access: 10.12.2023).

Increased fiber consumption can potentially reduce the risk of CRC through two non-mutually exclusive mechanisms. First, insoluble fiber accelerates colonic transit and may reduce exposure of colonic epithelial cells to ingested carcinogens such as heterocyclic amines from charred meat. Second, soluble fiber is fermented into butyrate and other potentially beneficial metabolites by certain classes of bacteria [29]. In a prospective study of stages I to III CRC patients, high grain-derived fiber intake was associated with lower CRC-specific mortality and overall mortality [30]. Consumption of a plant-based diet with a predominance of whole grains, fruits, and vegetables was associated with a lower incidence of CRC [28]. Fiber from bread and breakfast cereals was associated with a reduced risk of CRC [23].

It has been found that an increase in fish consumption of 20 g per day was associated with a 2% reduction in gastrointestinal cancer risk in a meta-analysis of 42 cohort studies, including 2,325,040 participants and 24,115 gastrointestinal cancer cases [31]. Yogurt and dairy-based desserts in women are negatively associated with the risk of CRC [27]. In a population of 923 CRC patients and 1846 healthy people, high coffee consumption (3 cups/day) was associated with a lower probability of developing CRC [32]. The relationship between diet, microbiota, and CRC is discussed later in the article.

## Microbiota and Its Functions

The microbiota plays an increasingly crucial role in the human digestive system, metabolism, immunity, and various other processes in the body, often referred to as another “organ” of the human body [33]. It has been estimated that close to 95% of these microbes are in the gut, especially the large intestine [https://internationalprobiotics.org/, Date of Access: 31.07.2023]. Considering the high bile concentrations and short transit time, the small intestine provides a more challenging environment for microbial colonization. In contrast, the colon, characterized by a neutral to slightly acidic pH and slow flow rate, hosts the largest microbial community [34].

The International Probiotic Association defines microbiota as follows: “The microbiota refers to the community of microorganisms, including bacteria, fungi, viruses, and yeasts, present in a defined environment and residing on or within human tissues such as the skin, lung, oral mucosa, and the urogenital and gastrointestinal tracts.” [https://internationalprobiotics.org/, Date of Access: 31.07.2023]. The human microbiota consists of bacteria, archaeabiota, virobiota, and micobiota, which form a highly complex network of interactions between each other and the host [35]. The human gastrointestinal system contains more than 100 trillion microorganisms. The density of bacterial cells is estimated to be 10^11^ to 10^12^ per milliliter in the colon. In Eubiosis, the predominant gut microbial phyla are Firmicutes, Bacteroidetes, Actinobacteria, Proteobacteria, Fusobacteria, and Verrucomicrobia. Firmicutes and Bacteroidetes phyla represent 90% of the gut microbiota. The Firmicutes phylum consists of more than 200 species, such as *Lactobacillus*, *Bacillus*, *Clostridium*, *Enterococcus*, and *Ruminicoccus*. The genus *Clostridium* represents 95% of the Firmicutes phylum. It consists of dominant genera such as Bacteroidetes, *Bacteroides*, and *Prevotella*. The phylum Actinobacteria is proportionally less abundant and is mainly represented by the genus *Bifidobacterium* [33]. Most current studies have focused on gut bacteria and neglected the mycobiota, archaeobiota, and virobiota. Chibani et al. [36•] determined 1167 archaeal genomes in human gastrointestinal tract samples. It has been determined NJ-bd that these genomes cover a wide taxonomic diversity and include members of the Methanobacteriales (87.15%), Methanomassiliicoccales (12.43%), Methanomicrobiales (0.26%), and Halobacteriales (0.17%). The predominant Archaea in the human gut are methanogens that anaerobically reduce carbon dioxide to methane gas. The most common methanogen in the gut microbiota is *Methanobrevibacter smithii*, with a prevalence of about 95.7% [37]. The human virobiota consists primarily of bacteriophages, animal cell viruses, endogenous retroviruses, and viruses that cause persistent and latent infections. Collectively, they contain a more diverse genetic entity than gut bacteria. A healthy human gut virome is predominated by temperate dsDNA Caudovirales and ssDNA Microviridae [38]. In a study examining fecal samples from 147 healthy volunteers, 701 fungal operational taxonomic units (OTUs) were identified, covering 247 genera. Specifically, fungal communities were characterized by a high prevalence of *Saccharomyces*, *Malassezia*, and *Candida*, with *S. cerevisiae*, *M. restricta*, and *C. albicans* OTUs [39].

Microbiota in early life, it has been shown to be affected by type of delivery [40], time of birth [41], breastfeeding, formula feeding [42], and pet ownership [43]. In the later stages of life, it has been determined that the microbiota is affected by diet, using drugs such as antibiotics and proton pump inhibitors [44, 45], stress [46], smoking, alcohol consumption [47], living in rural or urban areas [48], and physical activity [49].

The microbiota synthesizes numerous enzymes for digesting nutrients that escape the human digestive system. The gut microbiome is highly enriched for genes related to carbohydrate metabolism, including ≥ 115 glycoside hydrolase families and ≥ 21 polysaccharide lyase families [50]. Intestinal microbiota can also produce aspartic-, cysteine-, serine-, and metallo-proteases [51]. The microbiota biochemically modifies the substances taken up by the host, both exogenously and endogenously. Furthermore, they generate de novo metabolites [52]. Short-chain fatty acids (SCFAs) are mainly produced by saccharolytic fermentation of carbohydrates that escape digestion and absorption in the small intestine. SCFAs represent the primary carbon source from the diet to the host through the microbiome. The major SCFAs include formate, acetate, propionate, and butyrate. Additionally, amino acid fermentation contributes to SCFA production, mainly yielding acetate and propionate [53]. Butyrate constitutes the critical energy source for human colonocytes, which induces the proliferation of healthy colon cells while inducing apoptosis of colon cancer cells. Propionate directly supports energy homeostasis by reducing hepatic glucose production and adiposity. Acetate is an auxiliary metabolite necessary for the growth of other bacteria. Butyrate, propionate, and acetate show anti-inflammatory effects by regulating T-cell differentiation [54]. The microbiota plays a critical role in the maturation and regulation of the host’s immune functions. It prevents the proliferation of pathobionts and regulates colon cell proliferation and vascularization. It can synthesize vitamins and neurotransmitters, metabolize bile salts and xenobiotics, regulate mucus production, improve intestinal barrier integrity, and provide biotransformation of polyphenols [54–57]. Considering all these functions, it is not surprising that the microbiota is linked to metabolic, autoimmune, neurological, psychiatric, inflammatory, and cardiovascular diseases and cancer.

## Microbiota, Dysbiosis, Diet, and Their Relationship with CRC

**Fig. 1:**
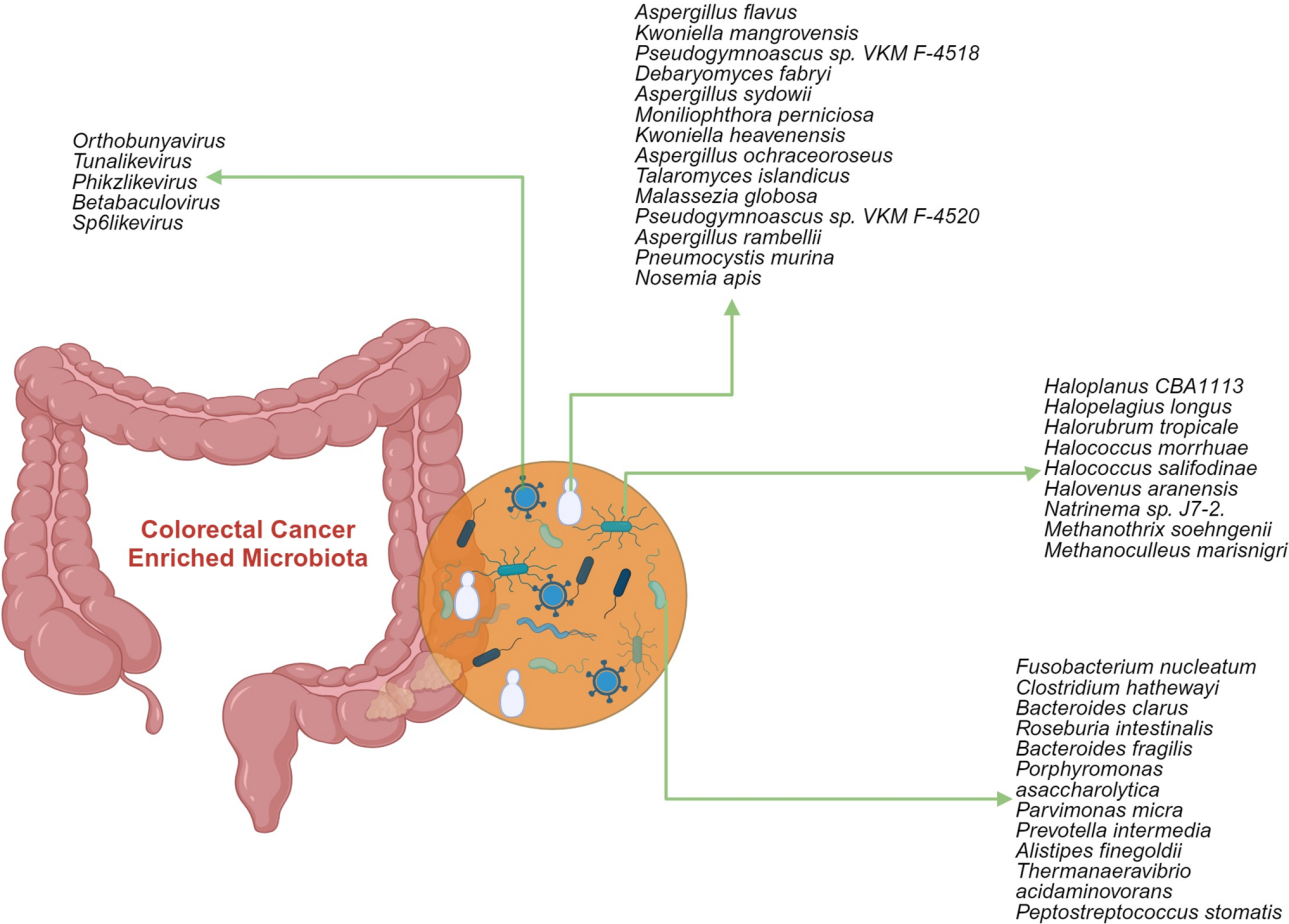
Colorectal cancer–enriched microbiota [59–66]

Dysbiosis is generally defined as a cause or consequence of a change and disorder in the gut microbiota composition. Dysbiosis is categorized into three types: (i) the loss of beneficial microbes, (ii) the proliferation of pathogenic microbes, and (iii) reduced microbial diversity [58]. It is often difficult to determine whether the change is beneficial or harmful [34]. CRC is linked to dysbiosis, which includes depletion and/or enrichment of certain intestinal bacterial species and their metabolic functions [14]. We summarized the microbiota identified to be enriched in colorectal cancer [59–66] in Fig. 1.

It is thought that holistic dysbiosis, rather than a specific pathogen in the microbiota, plays a role in the etiology of CRC [67]. The underlying mechanisms during the interaction between microbial dysbiosis and colorectal carcinogenesis include the promotion of inflammation, pathological bacterial adhesion, and induction of tumorigenesis [12]. It has been suggested that the microbiota differs between the early and late stages of CRC. Some pathogenic bacteria rapidly colonize the intestinal epithelium as drivers, while opportunistic microorganisms, namely travelers, further contribute to cancer progression [68]. It is stated that the changed microbiota in CRC can be used in diagnosis in the future and will contribute to the prevention/treatment of CRC [69]. Below, we summarize studies demonstrating changes in microorganisms and their metabolites in the analysis of rectal biopsy and fecal samples from CRC patients and healthy people.

In a study, biopsies were collected from normal rectal mucosa areas ∼10–12 cm from the anal verge in 33 adenoma subjects and 38 non-adenoma subjects, and bacterial genomic DNA was extracted from these biopsies. Biopsy samples from adenoma subjects showed higher relative abundance levels of 30 genera, including *Acidovorax*, *Aquabacterium*, *Cloacibacterium*, *Helicobacter*, *Lactococcus*, *Lactobacillus*, and *Pseudomonas*, compared to the controls. It has been determined that the bacteria, which were relatively high in the cases, were mostly members of the phylum Firmicutes, Bacteroidetes, and Proteobacteria. The authors suggest it can be used in future microbiota array analysis to identify patients at risk of developing adenomas [70]. In a study that included 203 CRC and 236 healthy controls, totaling 439 participants, higher relative abundances of *Fusobacterium nucleatum*, *Clostridium hathewayi*, *Bacteroides clarus*, and *Roseburia intestinalis* were observed in the fecal samples of CRC patients compared to the controls [59]. In a meta-analysis of 271 controls and 255 CRC cases, higher levels of *Bacteroides fragilis*, *Fusobacterium* nucleatum, *Porphyromonas asaccharolytica*, *Parvimonas micra*, *Prevotella intermedia*, *Alistipes finegoldii*, and *Thermanaeravibrio acidaminovorans* were detected in fecal samples from CRC cases compared to control [60].

In a study, microbiota and metabolomic profiling have been performed in fecal samples from 42 CRC cases and 89 controls. The results revealed strong microbe-metabolite correlations in CRC cases, primarily involving Enterobacteriaceae and Actinobacteria. CRC has been independently associated with lower levels of Clostridia, Lachnospiraceae, *p*-aminobenzoate, and conjugated linoleate and higher levels of *Fusobacterium*, *Porphyromonas*, *p*-hydroxy-benzaldehyde, and palmitoyl-sphingomyelin [71].

According to the results of a systematic analysis examining the relationship between gut microbiota and prognosis after CRC surgery, *Fusobacterium nucleatum* and *Bacteroides fragilis* have been associated with a worse prognosis [72].

In a systematic review including nine studies, organisms associated with CRC were identified as *Fusobacterium*, *Enterotoxigenic Bacteroides fragilis*, *Clostridium*, *Salmonella*, and *Peptostreptococcus* [73]. A meta-analysis of 57 studies demonstrated a higher presence of *Fusobacterium nucleatum* in tissue samples with CRC tumors [74].

As a result of the microbiome and metabolome analysis in fecal samples from 50 CRC patients and 50 healthy individuals, it has been determined that fecal samples of patients with CRC showed a unique and distinct metabolic signature. In CRC, there is a high presence of Proteobacteria, Fusobacteria, and lower levels of Firmicutes. In contrast, the control group exhibited higher levels of SCFAs and hydroxycinnamic acid. Additionally, CRC samples showed reduced microbial diversity. Fecal polyamines (cadaverine, putrescine) have been suggested as potential biomarkers for CRC due to their increased levels [67]. Additionally, CRC tissues have been found to be enriched with biofilms of *Bacteroides fragilis*, *Fusobacterium nucleatum*, *Parvimonas micra*, and *Peptostreptococcus stomatis* [75].

Fungal dysbiosis has been observed in patients with CRC and polyps. This dysbiosis is characterized by decreased fungal diversity, an increased Ascomycota/Basidiomycota ratio, and an elevated proportion of opportunistic fungi such as *Trichosporon* and *Malassezia*. These findings may contribute to the progression of CRC [61]. Biopsy was obtained from the adenoma and adjacent tissues of 27 subjects with colorectal adenomas. The analysis revealed a higher prevalence of the Glomeramycota phylum in adenomas [62]. As a result of the analysis of fecal shotgun metagenomic sequences of 184 patients with CRC, 197 patients with adenoma, and 204 control subjects, it has been determined that Basidiomycota-to-Ascomycota ratio was high, *Malasseziomycetes* increased, and *Saccharomycetes* and *Pneumocystidomycetes* decreased in CRC [63]. Archaeal dysbiosis has also been shown to occur in patients with CRC. It has been shown that methanogenic archaea are depleted, and halophilic archaea are enriched in patients with CRC [64]. A study examining fecal samples from three different cohorts showed that bacterial diversity in CRC patients decreased, the diversity of bacteriophage virome increased, and 22 viral taxa separated healthy controls from patients with CRC [65].

Genotoxic substances such as colibactin secreted by *Escherichia coli*, *Bacteroides fragilis* toxin produced by *Bacteroides fragilis*, and typhoid toxin from *Salmonella* can cause host DNA damage. These bacteria can also indirectly promote CRC by affecting host signaling pathways such as E-cadherin/β-catenin, Toll-like receptor-4 (TLR-4)/myeloid differentiation primary response protein 88 (MyD88)/nuclear factor kappa B (NF-κB), and smoothened (SMO)/rat sarcoma virus (RAS)/p38 mitogen-activated protein kinase (MAPK) pathway [76]. In dysbiosis, there is a decrease in the number of butyrate-producing bacteria such as *Bifidobacterium*, *Lactobacillus*, and *Bacteroides.* Simultaneously, there is an increase in the number of pathogenic bacteria such as *Clostridium difficile*, *Enterobacter*, *Fusobacterium nucleatum*, *Escherichia coli*, and *Klebsiella pneumonia.* These changes contribute to the deterioration of cell DNA structure, activation of carcinogenic signaling pathways, and induction of CRC [56]. Intestinal dysbacteriosis stimulates macrophage activation, which promotes the production and secretion of the inflammatory cytokines interleukin-6 (IL-6) and TNF-α (tumor necrosis factor-alpha), and elevated peripheral IL-6 and TNF-α subsequently support the epithelial-mesenchymal transition process of CRC that contributes to tumor progression and metastasis [77].

Microbiota has a bidirectional effect on CRC. The gut microbiota may support or protect against CRC through various mechanisms. The cancer-promoting microbiota adheres directly to epithelial cells via microorganism-associated molecular models and adhesins, such as Wingless-related integration site (Wnt)-β-catenin or oncogenic signal activation. It also produces toxins and harmful metabolites (such as secondary bile acids, polyamines, and hydrogen sulfide) and induces tumor-associated immune cell populations (such as tumor-associated macrophages, tumor-associated neutrophils, Myeloid-derived suppressor cells, and regulatory T cells) to regulate inflammation, tissue damage, cell proliferation and survival, immune evasion, and drug resistance. It can interact with the tumor microenvironment, affecting tumor growth and progression. In contrast, the gut microbiota may play a role in detoxifying dietary components and reducing chronic inflammation. Microbiota that inhibits CRC can directly trigger anti-tumor signal activation in epithelial cells, produce beneficial metabolites (such as SCFAs), and stimulate tumor-preventing immune cells (such as the cluster of differentiation-8 cells, T helper-1, T helper-17, and innate lymphoid cell-3) [69, 78].

Long-term dietary patterns exert a significant influence on our gut microbiome. However, short-term dietary interventions can also impact the gut microbiome [79]. The link between diet, gut microbiota, and CRC has primarily been established as an association rather than a cause-effect relationship. Microbiota can metabolize the gastrointestinal tract and dietary factors to oncometabolites and tumor suppressor metabolites [80]. Soluble fiber increases SCFA synthesis, exhibits prebiotic, anti-inflammatory, and antitumoral effects on microbiota, prevents dysbiosis, and enhances mucus secretion. On the other hand, insoluble fiber increases the rate of colonic transit and reduces the absorption of carcinogenic components. Thus, fiber strengthens the gut microbiota and potentially reduces the risk of developing colon cancer. Butyrate, one of the SCFAs, provides the necessary energy for the proliferation of colonic epithelial cells with rapid turnover. Colorectal tumor cells are exposed to the Warburg effect and switch to glucose utilization and aerobic glycolysis. Butyrate is not metabolized to the same extent and accumulates in the nucleus, which functions as a histone deacetylase (HDAC) inhibitor [81]. A meta-analysis of 17 case-controlled and six cross-sectional studies found that lower fecal SCFA concentrations were associated with a higher risk of CRC and incidence of CRC [82]. Normally, the amounts of proteolytic fermentation in the colon are smaller than those of saccharolytic fermentation. A high-fat, high-meat, low-fiber diet, pro-neoplastic, and pro-inflammatory properties of protein fermentation and bile acid deconjugated residues predominate, increasing CRC risk [83]. These diets produce pro-inflammatory and oncogenic components such as polyamines, ammonia, branched-chain SCFAs, nitrogen-rich metabolites, hydrogen sulfide (especially *F. nucleatum* and *Desulfovibrio vulgaris*), and secondary bile acids [84]. High-fat diets increase deoxycholic acid levels, which in turn resist apoptosis, trigger reactive oxygen species (ROS) generation and DNA damage, and activate NF-κB, ultimately increasing the risk of CRC [58]. In mice, dietary deoxycholic acid induced colonic tumors [85]. Trimethylamine-*N*-oxide (TMAO) is a microbiota-dependent metabolite from protein, particularly red meat, and a high TMAO level is associated with a higher risk of CRC. Firmicutes species contribute to TMAO production, while *Eubacterium limosum* has the potential to metabolize TMA precursors and reduce TMAO levels in the gut [78].

It is known that a diet rich in fruits, vegetables, and grains suppresses CRC, and not only high fiber content but also polyphenol content is effective in this regard [83]. Phytochemicals such as epigallocatechin-3-gallate, quercetin, ellagic acid, elicithins, proanthocyanidins, resveratrol, and curcumin can directly or indirectly affect the composition/metabolism of the gut microbiota and regulate gene expression epigenetically [86]. For example, the gut microbiota converts ellagitannins in foods such as pomegranates, strawberries, and nuts to a secondary polyphenol metabolite called urolithins. This urolithin has been shown to have anti-carcinogenic effects in CRC [87]. Thioglucosidases derived from the microbiota can convert glucosinolates found in vegetables such as broccoli and cabbage to isothiocyanates, which have anti-carcinogenic properties and act as HDAC inhibitors. The gut microbiota can conjugate linoleic acid [86]. Conjugated linoleic acid is reported to have anti-carcinogenic properties [88].

## Current Definitions and Information of Biotics (Probiotic, Prebiotic, and Postbiotic)

The critical relationship between gut microbiota, dysbiosis, and CRC, which we mentioned above, has pushed researchers toward investigating the effects of probiotics, prebiotics, and postbiotics in the prevention and treatment of CRC [15, 89]. The most current and widely accepted definition of probiotics, as articulated by Hill et al. [90], states that probiotics are live microorganisms that, when administered in adequate amounts, confer a health benefit on the host. The current scientific definition of the prebiotic is “A substrate that is selectively utilized by host microorganisms conferring a health benefit” [91].

The safety profile of probiotics in vulnerable and immunocompromised patients, the risk of developing antibiotic resistance and virulence gene transfer, the existence of problems with maintaining viability and stability during production and storage, and the demonstration that health benefits are not necessarily directly related to viable cells have led researchers to study of non-viable cells, especially heat-killed microorganisms, cell extracts, components, and metabolites [16, 92–94]. Before 2021, The International Scientific Association for Probiotics and Prebiotics (ISAPP) definition of postbiotics, researchers named them “cell-free supernatant,” “metabiotic,” “paraprobiotic,” “biogenic,” “ghost probiotics,” “abiotic,” “pseudoprobiotic,” and “postbiotic.” In 2019, ISAPP convened a panel of experts specializing in nutrition, microbial physiology, gastroenterology, pediatrics, food science, and microbiology to review and clarify the definition and scope of postbiotics. As a result of this panel, “the ISAPP consensus statement on the definition and scope of postbiotics” was published in 2021 [17••]. The committee defined a postbiotic as a “preparation of inanimate microorganisms and/or their components that confers a health benefit on the host.” Postbiotics may contain inanimated microbial cells, and/or cell components, with or without metabolites, that contribute to observed health benefits. Considering the data published by ISAPP, we have summarized the current definitions and important notes regarding probiotics, prebiotics, and postbiotics in Table 1.

**Table 1:** Current definitions and crucial notes with probiotics, prebiotics, and postbiotics [17, 90, 91]

	**Definition**	**Example**	**Crucial notes**
Probiotic	Live microorganisms that, when administered in adequate amounts, confer a health benefit on the host	• *Bifidobacterium animalis* subsp. *lactis* XYZ • *L. plantarum* ABC • *L. casei* 123	• Live microorganisms may be present in many foods and supplements but only characterized strains with a scientifically demonstrated effect on health should be called probiotics. • Probiotics are known by genus, species, and strain. • The dose should match the level shown in an efficacy study to confer a benefit.
Prebiotic	A substrate that is selectively utilized by host microorganisms conferring a health benefit	• İnulin • Fructooligosaccharides • Galactooligosaccharides • Human milk oligosaccharides	• Prebiotics are frequently equated with dietary fibers, but only a subset of dietary fibers qualify as prebiotics, and indeed, prebiotics may derive from non-fiber substances, such as polyphenols. • A prebiotic compound must confer a beneficial physiological effect on the host and that effect should derive at least in part from utilization of the compound by resident microbes.
Postbiotic	Preparation of inanimate microorganisms and/or their components that confer a health benefit on the host	• Postbiotics may contain intact inanimate microbial cells, and/or microbial cell fragments/structures (cell walls, membranes, exopolysaccharides, cell-wall anchored proteins, pili, etc.) with or without metabolites/end products (organic acids, peptides, secreted proteins, enzymes, bacteriocins, etc.)	• It must be derived from microorganisms. The postbiotic does not have to be derived from a probiotic. • A deliberate process must be applied to terminate cell viability. • The final postbiotic must contain inactivated microbial cells and/or metabolites or cell components. • Viable cells are absent or negligible in the final product. • There must be evidence of health benefits in the host from a controlled, high-quality trial. • A postbiotic must be safe for the intended use in the target host and this safety must be evaluated. • Viruses, bacteriophages, vaccines, purified microbial components (e.g., proteins, peptides, exopolysaccharides), filtrates without cell components, and purified microbial metabolites (e.g., organic acids) are not postbiotics.

ISAPP has determined the following issues regarding postbiotics. “Postbiotics do not need to be derived from a probiotic. The domain of action is not just the gut. Purified microbial metabolites, vaccines, and injections are beyond the scope of postbiotics. The host can include humans, companion animals, livestock and other targets. A postbiotic must be safe for the intended use in the target host and this safety must be evaluated. The procedure for inactivating the microorganism needs to be explained in detail and verified that inactivation of the microorganism has occurred. There must be evidence of health benefits in the host from a controlled, high-quality trial. The composition of the postbiotic preparation should be described in detail. The molecular characterization of progenitor microorganisms must be determined to ensure accurate identification and screening of potential genes of safety concern.” [https://isappscience.org/, Date of Access: 07.10.2023]. It is suggested that postbiotics are more advantageous than probiotics due to their stability in industrial processes and storage, long shelf life, not requiring a cold chain, more reliable profile in terms of health, and not having the risk of developing antibiotic resistance [14, 16, 69, 92–98].

## Postbiotics and CRC

**Fig. 2:**
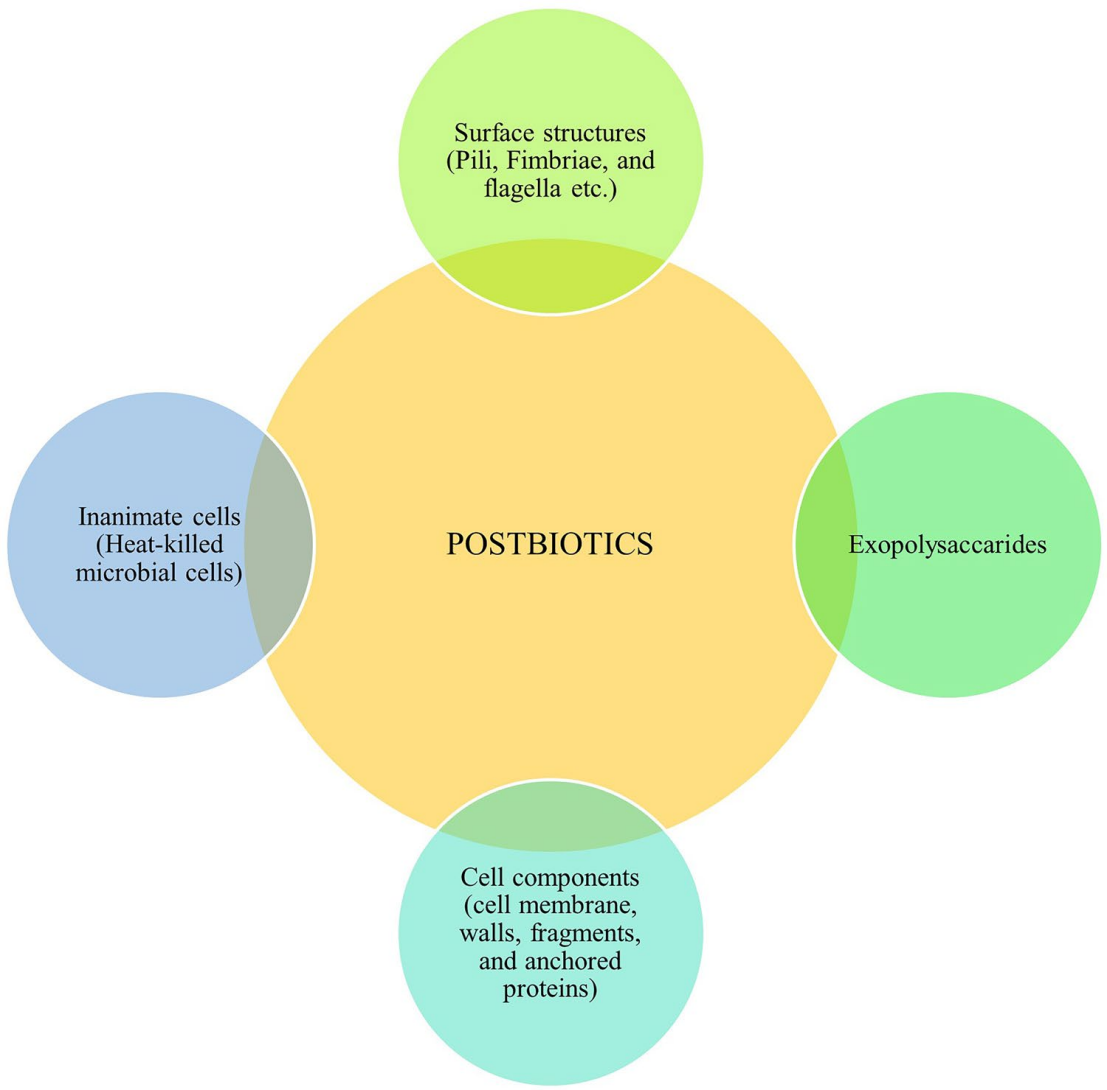
Types of postbiotics

We have collected the types of postbiotics in Fig. 2. The effects of postbiotics in CRC are discussed in detail below.

Techniques such as heat treatment, enzymatic processes, chemical treatments, sonication, hyperbaric conditions, and solvent extraction—either individually or in combination—are applied to inactivate microorganisms to produce postbiotics. Separating the desired postbiotics from the resulting solutions is subjected to steps such as centrifugation, dialysis, lyophilization, and column purification [92•].

### Heat-Killed Microorganisms and CRC

Heat-killed microorganisms were accepted as postbiotics by ISAPP in 2021 [17••]. Table 2 provides information about studies examining the relationship between heat-killed bacteria and CRC. In one study, some heat-killed *Bifidobacterium* and *Lactobacillus* strains have been shown to induce apoptosis in RKO cells, and administration of *L. casei MG4584* and *L. reuteri MG5346* (orally) to BALB/c nude mice xenografted with RKO cells significantly has been delaying tumor growth and inducing apoptosis in tumor tissues. Furthermore, when a mixture of *L. reuteri MG5346* and *L. casei MG4584* (Mix2) and a mixture of *B. bifidum MG731*, *L. reuteri MG5346*, and *L. casei MG4584* (Mix3) were administered to mice, the tumor inhibitory effect is enhanced, and tumor volume further is decreased [99].

**Table 2:** Studies on heat-killed microorganisms and colorectal cancer

**Microorganisms**	**Heat treatment**	**CRC model and treatment**	**Effects on CRC**	**References**
*Bifidobacterium bifidum MG731* *Bifidobacterium breve* *MG729* *Lactobacillus bulgaricus* MG515 *Lactobacillus casei* MG311 *Lactobacillus casei* MG4584 *Lactobacillus gasseri* MG4514 *Lactobacillus plantarum* MG4215 *Lactobacillus reuteri* MG5346 *Lactobacillus rhamnosus* MG316 *Lactobacillus rhamnosus* MG5200 *Streptococcus thermophilus *MG5140	100 °C, 30 min	RKO cells	• Cytotoxic effects: *Bifidobacterium bifidum MG731*, *Lactobacillus reuteri MG5346*, *Lactobacillus casei MG311*, *Lactobacillus casei MG4584*, and *Lactobacillus gasseri MG4514.* • Apoptosis induction: *Lactobacillus rhamnosus MG5200*, *Lactobacillus gasseri MG4514*, *Lactobacillus casei MG4584*, and* Lactobacillus casei MG311.*	[99]
		Xenografted BALB/c nude mice (RKO cells injected subcutaneously) Orally administered heat-killed bacterial strains	• Delay tumor growth, increase the expression of caspase 3,-7,-9, and PARP in tumor tissues: *Lactobacillus casei MG4584*, *Lactobacillus reuteri MG5346.*	
*Lactobacillus brevis* *Lactobacillus paracasei*	80 °C, 30 min	HT-29 cells	• Induce apoptosis. • Increase the expression of Bax, caspase-3–9. • Reduce the expression Bcl2 expression.	[100]
*Lactobacillus paracasei MPC2.1* *Lactobacillus GG*	95 °C, 60 min	HGC-27 cells DLD-1 cells	• Inhibition of the growth and proliferation (Both viable and heat-killed bacteria).	[101]
*Enterococcus faecalis*	80 °C, 30 min	DSS-induced colitis and colitis-associated CRC in mice Orally administered heat-killed bacterial strains	• Reduce the inflammatory activation of NLRP3 in macrophages. • Ameliorating the severity of colitis and the development of CRC.	[102]
*Mycobacterium paragordonae*	121 °C, 15 min	Xenograftic mice model (MC38 cells injected subcutaneously) Heat-killed mycobacteria were administered near the superficial inguinal lymph node	• Reduce tumor incidence, tumor progression, and tumor-related mortality. • Induce apoptosis. • Reduce the expression Bcl-2 and perforin. • Induce the cytotoxic ability of NK cells. • Synergistic effect cisplatin.	[103]
*Lactobacillus rhamnosus GG* *(Viable and heat-killed)*	80 °C, 20 min	5-FU-induced in vitro mucositis of Caco-2 cells	• Induce expression of TNF-α, MCP-1, IL-1 (both viable and heat-killed cells).	[104]
*Lactobacillus casei 1296–1* *Lactobacillus casei 1296–2* *Lactobacillus 1296–3* (Viable, heat-killed, CFS)	121 °C, 15 min	Caco-2 cells	• Cytotoxic effects: all strains. • Cytotoxicity effects of viable cells > heat-killed cells.	[105••]
*Lactobacillus plantarum A7* *Lactobacillus rhamnosus GG* (heat-killed and CFS)	95 °C, 60 min	Caco-2 and HT-29 cells	• Anti-proliferative activity: both heat-killed bacteria and CFSs. • Inhibit growth on cancer cell lines: CFS > heat-killed bacteria.	[106]

*5-FU* 5-fluorouracil, *BAX* Bcl-2-associated X protein, *Bcl-2* B-cell lymphoma 2, *CFS* cell-free supernatant, *DSS* dextran sodium sulfate, *IL-1* interleukin-1, *MCP-1* monocyte chemoattractant protein-1, *NLRP3* nucleotide-binding-domain- and leucine-rich repeat-containing family, pyrin-domain-containing 3, *NK* natural killer, *PARP* poly ADP ribose polymerase, *TNF-α* tumor necrosis factor-alpha. *Caco-2*, *DLD-1*, and *HT-29* human colorectal adenocarcinoma cell lines, *HGC-27* human gastric carcinoma cell line, *MC38* murine colon adenocarcinoma cells, *RKO* human colorectal carcinoma cell line

Heat-killed *Lactobacillus brevis* and *Lactobacillus paracasei* isolated from the traditional Iranian food “Terxine” have been shown to induce apoptosis in the HT-29 cell line. It has been shown that *L. brevis* has a greater ability to inhibit the growth of HT-29 cells and induce apoptosis, compared with *L. paracasei* [100].

Both viable and heat-killed *Lactobacillus paracasei MPC2.1* and *Lactobacillus GG* have been shown to inhibit the growth and proliferation of the human gastric carcinoma HGC-27 and colorectal adenocarcinoma DLD-1 cells. Based on these results, the authors suggested that dead probiotics may also be an effective dietary supplement [101].

Heat-killed cells of *Enterococcus faecalis* have been shown to effectively reduce the inflammatory activation of NLRP3 (nucleotide-binding-domain- and leucine-rich repeat-containing family, pyrin-domain-containing 3) in macrophages, ameliorating the severity of DSS (dextran sodium sulfate)-induced experimental colitis and the development of colitis-associated CRC [102].

In a xenograft mouse model in which MC38 cells derived from murine colon adenocarcinoma have been inoculated subcutaneously, administration of heat-killed *Mycobacterium paragordonae* by subcutaneous injection has reduced tumor incidence, tumor progression, and tumor-related mortality. It has induced apoptosis in tumor tissues and the cytotoxic ability of natural killer cells and inhibited tumor progression in a natural killer cell–dependent manner. Moreover, it has been shown that heat-killed *M. paragordonae* has a synergistic effect with the cancer chemotherapy drug cisplatin. Based on all the above results, the authors have considered that the potential use of heat-killed *M. paragordonae* as adjunctive immunotherapy can enhance the effect of chemotherapy [103]. In one study, it has been shown that heat-killed *Lactobacillus rhamnosus GG* remained structurally intact with elongation. Both live and heat-killed *L. rhamnosus GG* have been shown to induce expression of TNF-α, monocyte chemoattractant protein-1, and IL-1 genes on 5-fluorouracil (5-FU)-induced in vitro mucositis of Caco-2 cells. The authors have proposed that intestinal epithelium may be vulnerable to the post-chemotherapeutic use of live or dead *L. rhamnosus GG* in 5-FU-induced mucositis [104]. Although it is thought that the use of heat-killed microorganisms instead of probiotics is safer, this seems to be contradictory. More research is needed to elucidate this situation.

The strain, dose, viable and heat-killed state, exopolysaccharide (EPS), and other postbiotics of microorganisms can have different effects on CRC. For this reason, it is necessary to carry out more research on this subject and to provide an adequate database. Viable, heat-killed, and cell-free supernatant (CFS) of *Lactobacillus casei 1296-1*, *1296-2*, and *1296-3* strains showed cytotoxic effects on human colorectal adenocarcinoma cancer Caco2 cells. Moreover, the cytotoxicity effects of live cells on Caco2 cells were found to be significantly higher than on heat-killed cells. The authors suggested that live and CFS of *L. casei 1296-2* may be promising candidates for the CRC [105••]. Heat-killed cells and CFS of *Lactobacillus plantarum A7* and *L. rhamnosus GG* strain exhibited anti-proliferative activity on human colon cancer cell lines (Caco-2 and HT-29). In addition, CFS has been shown to further inhibit the growth of cancer cell lines [106]. The authors stated that this higher inhibitory effect of CFS could be attributed to the higher organic acid concentration in the supernatant, and neutralization may also need to be tried in the studies.

### Cell Components

Cell components are considered another important type of postbiotic. These may include plasma membrane, cell wall and its components (peptidoglycans), teichoic acid and lipoteichoic acid, surface layers (S-layers) proteins, organelles, flagella, pili, and capsules. Apart from mycoplasmas, both Gram-positive and Gram-negative bacteria have cell wall peptidoglycans that give the characteristic cell shape and provide mechanical protection to the cell. Peptidoglycan gets its name from its two main components: glycan strands made up of repeating disaccharide units and short peptide chains made up of two to five amino acid residues. The negatively charged teichoic acids found in Gram-positive bacteria encompass a diverse family of cell surface glycopolymers containing phosphodiester-linked polyol repeat units. Teichoic acids include both lipoteichoic acids that bind to the bacterial membrane via a glycolipid and wall teichoic acids that covalently bind to peptidoglycan [107, 108]. Table 3 provides information about studies examining the relationship between cell components and CRC.

**Table 3:** Studies on cell components and colorectal cancer

**Cell component-source**	**CRC model and treatment**	**Effects on CRC**	**References**
S-layer protein-*Lactobacillus acidophilus CICC 6074*	HT-29	• Induction of apoptosis. • Inhibiting PI3K/AKT pathway and synthesis of Fas Ligand.	[110]
Mucin binding protein->*Lactobacillus casei*	HT-29	• Anti-proliferative effects.	[111]
Cell wall***–***cytoplasmic extract, and nisin**-***Lactococcus lactis*	SW480	• Reducing cyclin D1 expression. • Anti-proliferative effect.	[112]
Cell wall*-L. acidophilus CL1285/L. casei LBC80R/L. rhamnosus CLR2*	HT-29	• Not affecting the growth of HT-29 cells. • When combined with cranberry fractions, inhibition growth.	[113]
Cell wall*-Saccharomyces boulardii*(gavage administration)	Rats on a high-fat low-fiber diet and treated with 1,2-DMH	• Reduced number of colon abnormal crypt foci. • Decreased b-glucuronidase activity. • Increased QR activity.	[114]
*Cell wall–*cytoplasmic extract*-Lactobacillus paracasei*subsp.* paracasei M5*	HT-29	• Dose-dependent cytotoxic effect. • Caused G2 phase arrest. • Altered the mitochondrial membrane potential. • Activated caspase 3. • Increased the cytosol cyto-c gene expression. • Increased the expression of BAX and BAD genes. • Decreased the expression of Bcl-xl gene.	[115]
The proteins (12 and 15 kDa)**-***Lactobacillus plantarum L67*(heat-killed)	HT-29	• Increased intracellular ROS and intracellular calcium and Bax and t-Bid. • Decreased cytochrome c, Bcl-2, caspase-8,-3, and PARP • Induction of apoptosis.	[116]
Cell wall and cytoplasm-*Lactobacillus X11, Lactobacillus K14, Lactobacillus paracaesi* subsp.*paracasei M5, Lactobacillus paracaesi* subsp.*paracasei X12*	HT-29	• Antiproliferative effects. • Induction of apoptosis. • DNA damage. • Decreased mitochondrial membrane potential.	[117]
Cell wall protein fractions-*Lactobacillus paracasei*	Caco-2	• Induction of apoptosis.	[118]

*Akt* a serine/threonine protein kinase, *BAD* BCL2-associated agonist of cell death, *Bax* B-cell lymphoma 2-associated X protein, *Bcl-2* B-cell lymphoma 2, *Bcl-xl* B-cell lymphoma-extra-large, *PARP* poly ADP ribose polymerase, *PI3K* phosphatidylinositol 3-kinase, *QR* quinone reductase, *t-Bid* truncated BH3 interacting domain death agonist. *Caco-2*, *HT-29*, *SW480* human colorectal adenocarcinoma cell lines

Monomolecular arrays of protein or glycoprotein subunits that make up the S-layers are one of the most frequently observed prokaryotic cell envelope charges [109]. S-layer protein obtained from *Lactobacillus acidophilus CICC 6074* has been shown to induce apoptosis on HT-29 cells and inhibit the phosphatidylinositol 3-kinase (PI3K)/a serine/threonine protein kinase (AKT) pathway and Fas-Ligand synthesis [110].

It has been shown that mucin-binding protein obtained from *Lactobacillus casei* can inhibit proliferation in HT-29 cells [111]. It has been determined that cytoplasmic extract and cell wall of *Lactococcus lactis*, and nisin showed antiproliferative effects by reducing cyclin D1 expression in SW480 cells. Nisin has shown the highest antiproliferative effect, followed by cytoplasmic extract and cell wall, respectively [112].

A cell wall extract from *Lactobacillus acidophilus CL1285*, *L. casei LBC80R*, and *L. rhamnosus CLR2* has not affected the growth of HT-29 colon cancer cells. However, when combined with cranberry fractions, a significant increase in inhibition rate has been observed. In this study, the authors stated that probiotic components and phenolic compounds in foods may act synergistically in colon cancer [113].

Gavage administration of insoluble glucan extract obtained from *Saccharomyces boulardii* cell wall in rats on a high-fat, low-fiber diet and treated with 1,2-dimethylhydrazine has been shown to reduce the number of colon abnormal crypt foci, decrease *b*-glucuronidase activity, and increase nicotinamide adenine dinucleotide phosphate (NADPH):quinone reductase activity in cecum [114].

It has been shown that the *Lactobacillus paracasei* subsp. *paracasei M5* strain obtained from Chinese traditional koumiss has a dose-dependent cytotoxic effect on HT-29 cells of the cell wall, causes G2 phase arrest, alters the mitochondrial membrane potential, activates caspase 3, increases Cyto-C gene expression in the cytosol, increases the expression of BAX- and BCL2-associated agonist of cell death (BAD) genes, and decreases the expression of B-cell lymphoma-extra-large (Bcl-xl) gene [115]. Proteins (12 and 15 kDa) from heat-killed *Lactobacillus plantarum L67* have been shown to induce apoptosis in HT-29 cells [116].

Wang et al. [117] obtained a total of 138 *Lactobacillus* strains from conventional fermented foods and infant feces. They showed that 10 strains had higher anti-proliferative activity and higher adhesion ability on HT-29 cells. They then screened these strains for resistance to acid and bile salts and selected the four most promising strains. The cell walls and cytoplasm extracts of these 4 strains have been shown to cause antiproliferative activity and DNA strand breakage on HT-29 cells. Cell walls extracted from strains X12, M5, and K14 and cytoplasm from strain M5 have been shown to disrupt mitochondrial membrane potential and induce apoptosis in HT-29 cells. However, it was determined to be less harmful to non-cancerous Vero cells than to human colon cancer HT-29 cells.

### EPSs and CRC

EPSs are extracellular high-molecular-weight biopolymers composed of sugar residues secreted by a microorganism into the surrounding environment during their growth [119]. EPSs are homopolysaccharides or heteropolysaccharides with different properties in terms of their composition, structural conformation, molecular weight, and functional groups. Microbial EPSs are more cost-effective than polysaccharides of plant and animal origin, and large amounts of EPS can be produced in a short time using low-cost substrates such as microorganism wastes [120].

Besides protecting probiotic organisms against harsh environmental conditions, they also take part in cell recognition and biofilm formation. They play the most prominent role against desiccation, phagocytosis, cell recognition, phage attack, antibiotics or toxic compounds, and osmotic stress. EPS can form a loosely attached layer or be secreted into the extracellular area. In the last few decades, natural polymers have gained much attention among scientific communities owing to their therapeutic potential [121, 122]. EPS is not catabolized by the human digestive system and enters the cecum and colon, where the microbiota ferments EPS to produce beneficial substances, especially SCFA, also lowers pH, inhibits the growth of pathogens, increases the abundance of beneficial bacteria, provides energy for colonic epithelial cells, and increases intestinal barrier function [123]. Studies examining the relationship of EPS with CRC are increasing day by day. Various studies have examined the effect of microbial EPS on CRC (Table 4).

**Table 4:** Studies on exopolysaccharides and colorectal cancer

**Source of EPS**	**Content of EPS**	**Obtaining method of EPS**	**CRC model and treatment**	**Effects on CRC**	**References**
*Lactiplantibacillus plantarum 12* (oral intake)	Galactose, mannose, glucuronic acid, galactosamine, glucose, and xylose	Cell culture-centrifugation to obtain cell-free supernatant-boiling-concentration by evaporation-TCA addition and mixing-cold ethanol addition and centrifugation-suspension with deionized water-dialysis-lyophilization	C57BL/6 mice treated by AOM/DSS	• Alleviating colon cancer symptoms. • Modulating microbiota, and metabolites. • Strengthening the gut barrier. • Inhibiting the NF-κB and p38 MAPK pathway. • Activating the caspase sequence.	[123]
*Levilactobacillus brevis LB63* *Lactiplantibacillus plantarum GD2* *Lacticaseibacillus rhamnosus E9* (Both viable cells and EPS)	ND	Incubation-TCA treatment-mixing-centrifuge-evaporation-ethanol precipitation-centrifuge-suspension with ultrapure water-lyophilization	HT-29	• Anti-genotoxic, immunomodulator, anti-proliferative effects (both viable cells and EPS). • The effects of L-EPSs were close to viable cells. • Best anti-genotoxic effects: viable cells (especially *Levilactobacillus brevis LB63*) and EPS (E9).	[124]
*Lactobacillus acidophilus* (oral intake)	ND	Incubation-TCA treatment-incubation-centrifugation, addition of ethanol to the supernatant-incubation-dissolution of precipitate with distilled water-extraction with cold ethanol-dialysis	Rats with DMH-induced colon cancer	• Reduction in the number of polyps. • Restoring the levels of antioxidative enzymes (SOD, CAT, GPx) and vit C.	[125]
*Rhizopus nigricans* (oral intake)	Glucose, mannose, galactose, and fructose at a molar ratio of 5.89:3.64:3.20:1.00, respectively [127]	Incubation, concentration by evaporation-treatment with ethanol, deproteinization-decolorization with D301R resin-dialysis-lyophilization-elution with NaCl solution-purification with Sephadex G-100 and Sephadex G-50 column-elution with distilled water-dialysis-lyophilization	CT26 cells	• Growth suppression. • Induction of apoptosis by AMPK pathway activation, mTORC1 inhibition, JNK-p53 activation and increase in caspase-3 expressions.	[126]
			CRC mice treated with AOM/DSS	• Activation of the AMPK pathway, inhibition of mTORC1, and p53 accumulation in tumor tissues.	
*Kluyveromyces marxianus* *Pichia kudriavzevii*	ND	Isolation-incubation-centrifuge-added isopropanol to the supernatant-centrifuge-dissolved in distilled water-lyophilization	SW-480 cells HT-29 cells HCT-116 cells	• Reduce the cells viability. • Induction of apoptosis. • Inhibition of proliferation of cancerous cell lines similar to 5-FU. • Lower cytotoxic effect in KDR/293 cells compared to 5-FU. • Increased expression of proapoptotic genes (BAX, caspase-3 and -8) and decreased anti-apoptotic gene (Bcl-2), AKT-1, JAK-1 and mTOR gene.	[128]
*Lactobacillus plantarum GD2, Lactobacillus rhamnosus E9, Lactobacillus brevis LB63, Lactobacillus delbrueckii* ssp. *bulgaricus B3*	All EPSs contain different relative proportions of mannose, glucose and *N*-acetylglucosamine. Some contain arabinose or fructose	Isolation-incubation-TCA treatment-centrifugation, ethanol precipitation-centrifugation-suspension of the pellet in water-lyophilization	HT-29 cells	• Antiproliferative effect. • Decrease in Bcl-2 and survivin expression. • Increase in BAX, caspase 3 and 9 expressions. • Induction of apoptosis. • EPS of *L. delbrueckii* ssp. *bulgaricus* B3 showed the highest induction of apoptosis.	[129]
*Lactobacillus kefiri*	Heteropolysaccharide with a repeating unit containing glucose and galactose	Incubation-boiling-centrifuge-TCA in cell-free culture-incubation-centrifuge-ethanol precipitation-incubation-centrifuge-dissolution in distilled water-dialysis-lyophilization	HT-29 cells	• Anticancer effect. • Up-regulate the expression of BAX, BAD, caspase 3, caspase 8, and caspase 9. • Down-regulate the BCl-2.	[130]
***Lactobacillus casei M5*** ***Lactobacillus casei SB27*** *Lactobacillus casei* × *12* *Lactobacillus casei *K11	ND	Isolation-incubation-pH reduction-boiling-centrifugation-filtration-pH adjustment with NaOH-lyophilization, purification by anion exchange chromatography, collection	HT-29 cells	• Antiproliferative effect (via G0/G1 cell cycle arrest in the cell cycle. • Increasing caspase 3 activity. • Best inhibitory effect: acidic EPS produced from SB27. • Not toxic to Vero cells.	[131]
*Lactobacillus acidophilus 20079*	*α-D-Glc(1 → 2)][α-L-Fuc(1 → 4)] α-D-GlcA(1 → 2) α-D-GlcA(1 → 2) α-D-GlcA*	Incubation-TCA treatment-centrifugation-alcohol extraction-recovery by centrifuge-evaporation-dialysis-purification by DEAE-cellulose column	CaCo-2 cells	• Cell viability inhibition. • Induction of apoptosis via sub G0/G1 cell cycle (arrest in the cell cycle). • Up-regulation of IKba, P53, and TGF gene expression.	[132]
*Lactobacillus plantarum* 70810	α-d-(1 → 2,3)-linked galactcosyl residues and a tail end of β-d-(1 →)-linked galactcosyl residues	Incubation, centrifuge, washing with NaCl solution, centrifugation Then 3 different methods (A,B,C): Method A: Resuspension in NaCl and cell sonicator, centrifugation, mixing with alcohol, incubation, precipitate collection, centrifugation, dialysis, lyophilization Method B: Resuspension and shaking in 0.5% phenol, centrifugation, mixing with alcohol, incubation, precipitate collection, centrifugation, dialysis, lyophilization Method C: Suspension and mixing in EDTA, centrifugation, mixing with alcohol, incubation, precipitate collection, centrifugation, dialysis, lyophilization Maximum yield (64.17 mg/mL) was obtained by method A	HepG-2 cells BGC-823 cells HT-29 cells	• Inhibited the proliferation (especially HT-29 tumor cells).	[133]
*Lactobacillus casei *01	The main constituent of the EPS molecules: galactose (70% w/w), and glucose (25% w/w)	Bacteria incubation-centrifugation of cells and obtaining cell-free culture supernatant-centrifugation with chilled ethanol-freeze-drying of the pellet and suspension in PBS	HT-29 cells	• Antiproliferative effect. • Reduce the cytotoxic effect of 4-NQO on intestinal-407 cells.	[134]

*4-NQO* 4-nitroquinoline-1-oxide, *5-FU* 5-fluorouracil, *AKT-1* Akt serine/threonine kinase 1, *AMPK* AMP-activated protein kinase, *AOM/DSS* azoxymethane/dextran sulfate sodium, *BAD* BCL2-associated agonist of cell death, *BAX* Bcl-2-associated X protein, *Bcl-2* B-cell lymphoma 2, *CAT* catalase, *DMH* 1,2-dimethyl hydrazine, *EDTA* ethylenediaminetetraacetic acid, *EPS* exopolysaccharide, *GPx* glutathione peroxidase, *IKba* IkappaB kinase alpha, *JAK-1* Janus kinase 1, *JNK* Jun-N-terminal kinase, *mTORC1* mammalian target of rapamycin complex 1, *NF-κB* nuclear factor kappa B, *p38 MAPK* p38 mitogen-activated protein kinases (MAPKs), *SOD* superoxide dismutase, *TCA* trichloroacetic acid, *TGF* transforming growth factor. *Caco-2*, *HCT-116*, *HT-29*, *SW480* human colorectal adenocarcinoma cell lines; *BGC-823* human gastric cancer cell line; *CT26* murine colon cancer cell line. *HepG-2* human liver cancer cell line

In another study, LP-EPS produced by *Lactiplantibacillus plantarum*-12 was administered orally daily for 85 days to C57BL/6 mice treated with azoxymethane (AOM)/DSS salt. LP-EPS supplementation increased colon tight junction protein (Claudin-1) expressions, increased goblet cell number, restored crypt structure, significantly decreased serum proinflammatory cytokines TNF-α, IL-8, and IL-1β levels, and increased anti-inflammatory factor IL-10. The authors have suggested that this LP-EPS could be used as a potential active ingredient to alleviate inflammation and colon cancer burden in colon cancer patients [123].

Both viable cells and EPSs of *Levilactobacillus brevis* LB63, *Lactiplantibacillus plantarum* GD2, and *Lacticaseibacillus rhamnosus* E9 bacteria have shown anti-genotoxic, immunomodulatory, and anti-proliferative effects on HT-29. Especially viable LB63 strain and EPS of E9 have been found to have anti-genotoxic activity. It has been found that the effects of EPSs were like viable cells. Based on these results, the authors suggested that live probiotics and EPSs could be natural anti-cancer agents for CRC [124].

Oral administration of EPS obtained from *Lactobacillus acidophilus* on rats that developed 1,2-dimethyl hydrazine–induced colon cancer decreased the number of polyps, restored the levels of antioxidative enzymes (superoxide dismutase, catalase, and glutathione peroxidase) and vitamin C to near normal levels, which decreased in the colon during carcinogenesis [125].

It has been shown that EPS1-1 isolated from *Rhizopus nigricans* induced apoptosis in murine colon cancer CT26 cells and tumor tissues of CRC mice treated with AOM/DSS. Based on these findings, the researchers have suggested that EPS1-1 may be a potential anti-CRC drug [126]. In the study of Saadat et al. [128], EPSs from *Kluyveromyces marxianus* and *Pichia kudriavzevii* were applied to colon cancer cell lines (SW-480, HT-29, HCT-116) and normal cell line (KDR/293). EPS application has reduced cell viability in colon cancer cell lines, induced apoptosis, inhibited the proliferation of cancer cells like 5-FU, and increased the expression of proapoptotic genes (BAX, caspase-3–8) and anti-apoptotic gene Bcl-2 and the expression of AKT-1, Janus kinase 1, and mTOR genes. A lower cytotoxic effect has been seen in KDR/293 cells compared to 5-FU.

In one study, EPSs have been obtained from 4 strains isolated from healthy infant feces (*Lactobacillus plantarum GD2*, *L. rhamnosus E9*, *L. brevis LB63*) and yogurt (*L. delbrueckii* ssp. *bulgaricus B3*). These EPSs have been shown to inhibit proliferation and induce apoptosis in HT-29 cells. EPS produced from *L. delbrueckii* ssp. *bulgaricus B3* has had the highest mannose and lowest glucose and this EPS showed the highest induction of apoptosis. Researchers have demonstrated a relationship between the ability of EPSs to induce apoptosis and the composition of mannose and glucose [129].

MSR101, EPS produced by *Lactobacillus kefiri* isolated from Chinese kefir grains, has been shown to have an anticancer effect on HT-29 cells and up-regulate the expression of BAX, BAD, and caspase3-8-9 and down-regulate BCl-2 [130].

EPS obtained from *Lactobacillus casei M5*, *L. casei SB27*, *L. casei* × 12, and *L. casei K11*, especially acidic EPS produced by *L. casei* SB27, induced apoptosis via caspase-3 activation, and they have been not toxic effects in Vero cells [131]. LA-EPS-20079 produced by *L. acidophilus* 20,079 has been shown to inhibit cell viability, induce apoptosis (via sub G0/G1 cell cycle arrest in the cell cycle), and up-regulate the expression of IkappaB kinase alpha, P53, and transforming growth factor in Caco-2 cells [132].

It has been shown that cell-bound EPS produced by *Lactobacillus plantarum* 70810 significantly inhibits proliferation in HepG-2, BGC-823, and especially HT-29 tumor cells. In addition, the authors showed that EPS production method differences may affect EPS yield [133]. The effects of *L. casei* 01 on HT-29 and intestine-407 cell proliferation, including its heat-inactivated form, cell wall, intracellular extracts, and EPSs, have been investigated. The highest antiproliferation activity was demonstrated by EPSs. It has also been shown that these EPSs reduce the cytotoxic effect of 4-nitroquinoline-1-oxide on intestinal-407 cells [134].

### Other Substances Produced by Microorganisms, Not Included in the Definition of Postbiotic, but May Be Present in Postbiotics, and Their Relationships with CRC

Postbiotics must include inanimate microorganisms and/or cell components. These substances may or may not contain metabolites. However, it is essential to note that purified metabolites are not considered postbiotic [17••]. In this part of our review, we focus on CFS and metabolites (enzymes, bacteriocins, SCFA, and others) produced by microorganisms that can be found in postbiotics, although they are no longer considered postbiotics and their relationship with CRC.

#### CFS and CRC

CFS is obtained from bacterial cultures after the incubation step, by centrifuging the culture medium, removing the pellet, and filtering the supernatant. CFS contains metabolites from microbial growth and residual nutrients of the medium used. It is a complex of enzymes, proteins, SCFA, vitamins, surfactants, amino acids, peptides, organic acids, and metabolic products secreted into CFS [18, 135]. A summary of studies showing the effects of CFSs on CRC is given in Table 5.

**Table 5:** Studies on cell-free supernatant and colorectal cancer

**Source of CFS**	**CRC Model and Treatment**	**Effects on CRC**	**References**
*Lactobacillus rhamnosus GG*	• HCT-116, Caco-2, and HT-29 cells • A375 cells	• Decreased cell viability. • Mitotic arrest in the G2/M phase of the cell cycle. • Selectively reduced the viability of cancer cells. • No anti-proliferative activity on control fibroblasts. • A positive synergistic effect by sensitizing cancer cells to both 5-FU and irinotecan.	[136]
*Lacticaseibacillus paracasei SD1* *Lacticaseibacillus rhamnosus SD4* *Lacticaseibacillus rhamnosus SD11* *Lacticaseibacillus rhamnosus GG*	• Caco-2 cells	• Inhibited growth. • Inhibited expressions of IL-1β, IL-6, IL-8, and TNFα. • Stimulated the expression of hBD (2–4) and IL-10.	[137]
*Odoribacter splanchnicus*	• HCT-116, CT 26 cells • CCD841CoN cells	• Inducted of apoptosis. • Inhibition of proliferation. • No cytotoxic effects on CCD841CoN cell line.	[138]
	CRC mice allograft model (CT 26 cells were inoculated subcutaneously into the flanks of BALB/c mice)	• Reduced tumor growth, volume, and weight.	
*Lactobacillus reuteri* (Both CFS and heat-killed sonicated bacteria)	HT29-ShE cells	• Reduced the cell invasion. • Decreased the level and activity of MMP-9. • Increased the level of TIMP-1. • Induced of apoptosis.	[139]
*Lactobacillus fermentum* CFS	3D spheroids of HT-29, DLD1, and WiDr	• Induces apoptosis. • Increases BAX, BAK and NOXA expression. • Decreases PARP-1 and BCL-XL expression. • Inhibits NF-κB.	[140]
*Lactobacillus rhamnosus MD14*	Caco-2 and HT-29 cells	• Genotoxic and cytotoxic effects. • Mitotic arrest in the G0/G1 phase of the cell cycle.	[141]
*Lactobacillus rhamnosus GG* *Lactobacillus casei M3* *Lactobacillus plantarum YYC-3*	Caco-2 and HT-29 cells	• Inhibited cell growth, invasion, and migration. • Inhibited MMP-2 and -9. • Down-regulated VEGF-MMP signaling pathway. • Like that of 5-FU effects.	[142]
*Lactobacillus plantarum YYC-3* (Both CFS and viable cell) (oral administration)	APCMin/ + mice	• Reduced colon tumors and mucosal damage. • Modulated the immune system. • Reduced the infiltration of inflammatory cells. • Reduced IL-6, IL-17 F, IL22, b catenin, Myc, cyclin-D1, Vcam1, Icam1. • Suppressed NF-κB and Wnt signaling pathways. Improved intestinal dysbiosis.	[143]
*Lactobacillus plantarum*	5-FU chemotherapy-resistant HT-29 and HCT-116 cells	• Reduced the CD44, CD133, CD166, and ALDH1. • Inhibited proliferation. • Inhibited Wnt/β-catenin pathway. • Inhibited formation of the colonosphere. • Increased apoptosis and caspase-3 activation, together with 5-FU.	[144]
*Enterococcus lactis IW5*	Caco-2, HeLa, MCF-7, AGS, HT-29, FHs-74	• Decreased the viability of cancer cells. • No effect on viability of normal FHs-74 cells. • Strongly adhered to cells.	[145]
*Lactobacillus casei,* *Lactobacillus rhamnosus*	HCT-116	• Reduced cell invasion. • Decrease MMP-9. • Increase Zo-1.	[146]

*5-FU* 5-fluorouracil, *ALDH1* aldehyde dehydrogenase 1, *BAK* Bcl-2-antagonist/killer, *BAX* Bcl-2-associated X protein, *CD* cluster of differentiation, *hβ* human β-defensin, *Icam1* ıntercellular adhesion molecule 1, *IL* interleukin, *MMP-9* metalloproteinase-9, *Noxa* BH3-containing mitochondrial protein, *NF-κB* nuclear factor kappa, *TIMP-1* tissue inhibitor of metalloproteinases 1, *TNF-α* tumor necrosis factor alpha, *Vcam1* vascular cell adhesion molecule 1, *Zo-1* Zona occluded-1, *A375* human malign melanoma cell line, *AGS* human gastric cancer cells, *Caco-2*, *DLD-1*, *HCT*-*116*, *HT*-*29*, *WiDr* human colorectal adenocarcinoma cell lines, *CCD841CoN* human normal colon cell, *CT26* and *MC38* murine colon cancer cells, *FHs-74* human small intestinal epithelial cell line, *HeLa* human cervix adenocarcinoma cells, *HGC-27* human gastric carcinoma cell line, *HT29-ShE cells* human colon cancer stem-like cells, *MCF-7* human breast cancer cell line

Salemi et al. [136] showed that the CFS of *Lactobacillus rhamnosus GG* reduced the viability of HCT-116, Caco-2, HT-29 (human colon cancer cell lines), and A375 (malignant melanoma cell line). CFS has been shown a positive synergistic effect by sensitizing cancer cells to both 5-FU and irinotecan. The authors have indicated that they propose CFS of *L. rhamnosus GG* as an ideal candidate for improving therapeutic response in cancer patients.

CFSs and cell walls of *Lacticaseibacillus paracasei SD1*, *L. rhamnosus SD4*, *SD11*, and *GG* inhibited the release of IL-1β, IL-6, IL-8, and TNFα; stimulated the expression of human β-defensin -2, -4, and IL-10; and inhibited growth in Caco-2 cells [137].

In a study, CFSs have been prepared from 100 gut microorganisms isolated from human feces, and 21 of them have been shown to have antiproliferative effects in HCT-116 cells. Among these CFSs, the CFS of *Odoribacter splanchnicus* has been identified as exhibiting the highest anti-cancer activity in both HCT-116 and CT 26 mice colon cancer cells. Active molecule believed to be present in this CFS has been suggested to be malic acid. Additionally, in the CRC mice allograft model, peri-tumoral injection of this CFS has demonstrated a reduction in tumor growth, volume, and weight [138].

It has been shown that the heat-killed (100 °C for 2 h) sonicated fraction and CFS of *Lactobacillus reuteri* reduced the invasion and induced apoptosis in HT29-ShE cells. It has been suggested that these effects arise from secretory macromolecules such as polysaccharides, nucleic acids, or proteins [139].

CFS of *Lactobacillus fermentum* induces apoptosis in three-dimensional (3D) spheroids of HT-29, DLD1, and WiDr; increases BAX, BAK (Bcl-2 homologous antagonist/killer), and BH3-containing mitochondrial protein; decreases PARP (poly ADP ribose polymerase)-1 and BCL-XL expressions; and inhibits NFκB [140].

Among the CFSs of 60 *Lactobacillus* strains isolated from different sources, it has been determined that the CFS with the highest antigenotoxicity and cytotoxicity on Caco-2 and HT-29 cells belonged to *L. rhamnosus MD14*. As a result of the physicochemical characterization of CFS, the presence of heat-sensitive organic acids and proteins has been demonstrated [141]. Yue et al. [142] have found that CFSs of *Lactobacillus rhamnosus* GG, *L. casei* M3, and *L. plantarum* YYC-3 inhibit growth, invasion, migration, and cell metastasis in Caco2 and HT-29 cells. Moreover, they have determined that this inhibitory effect of all CFSs was like that of 5-FU.

It has been shown that oral administration of both the *Lactobacillus plantarum* YYC-3 strain and its CFS to APCMin/+ mice fed a high-fat diet prevented the formation of colon tumors and mucosal damage, modulated the immune system, reduced the infiltration of inflammatory cells, and improved the gut dysbiosis [143].

It has been shown that CFS of *Lactobacillus plantarum* inhibits proliferation, Wnt/β-catenin pathway, and colonosphere formation in HT-29 and HCT-116 cells resistant to 5-FU chemotherapy and increases apoptosis and caspase-3 activation when applied together with 5-FU [144].

Nami et al. [145] showed that CFS of *Enterococcus lactis* IW5 from the human intestine strongly decreased the viability of different cancer cells such as HeLa, MCF-7, AGS, HT-29, and Caco-2 and did not prevent the viability of normal FHs-74 cells.

CFS of *L. casei* and *L. rhamnosus* has been determined to reduce the invasion of HCT-116 cells. It has been suggested that the effective compound may be a macromolecule such as a protein, nucleic acid, or polysaccharide [146].

#### Metabolites

Plantarisin BM-1, a bacteriocin produced by *Lactobacillus plantarum*, significantly reduces the viability of SW480, Caco-2, and HCT-116 CRC cells, with a more pronounced impact on SW480 [147]. In a study that screened 16,000 bacterial clones from human fecal samples, salivaricin A5 and salivaricin B 243, the bacteriocins of *Streptococcus salivarius*, were shown to have selective antimicrobial activity against pro-tumorigenic *Fusobacterium nucleatum* strains and are a biotherapeutic that can target pathogens causing CRC [148]. Reuterin produced by *Lactobacillus reuteri* has been shown to inhibit colon tumor growth in both in vitro and in vivo settings by modifying the redox balance, inducing protein oxidation, and inhibiting ribosomal biogenesis [149]. Indole-3-lactic acid produced by *Lactobacillus gallinarum* has significantly reduced tumor number and size in APC Min/+ mice [150].

Mice receiving orally catalase-producing *Lactococcus lactis* have been shown to have significantly less colonic damage and inflammation and prevent tumorigenesis compared to animals not receiving catalase-producing *L. lactis* or bacterial supplementation in an experimental DMH-induced colon cancer mouse model [151]. It has been reported that β-galactosidase secreted by *Streptococcus thermophilus* can prevent colon tumor formation in mice injected with APC min/+ and AOM [152].

It has been shown that SCFA obtained from *L. reuteri* induces apoptosis in HT-29 cells [153]. Among the SCFAs, butyrate is the most potent in inhibiting HDAC activities both in vitro and in vivo [154]. Butyrate inhibits glucose uptake and membrane abundance of glucose transporter 1 (GLUT-1) in HCT116 and LoVo cells, decreases AKT phosphorylation, and decreases the expressions of ribose-5-phosphate, acetyl-CoA, NADPH, and glucose-6-phosphate dehydrogenase. It has been determined that it inhibits DNA synthesis and has a synergistic effect with 5-FU [155]. Butyrate has been shown to inhibit the invasion of HCT116, HT29, LOVO, and HCT8 CRC cells [156]. In the AOM/DSS-induced CRC mouse model, gavage-ingested butyrate has been reported to modulate the microbiota, attenuate weight loss, disease activity index, and survival and inhibit tumor number and progression [157]. Sodium butyrate has been shown to induce apoptosis in HT29 cells [158]. Butyrate has been shown to increase ROS levels, inhibit growth, decrease cyclin D2 protein levels, and increase P21 and cleaved PARP levels in LT97 and HT29 colon cells [159]. It has been shown that acetate reduces proliferation, decreases glycolysis, and increases oxygen consumption and ROS levels in HT29 and HCT116 colon cancer cells [160].

## Future Perspectives

Postbiotics are still in their infancy and more studies need to be done in the future. Some of those: Inactivation steps and techniques of microorganisms should be standardized. The composition of the postbiotic product should be defined. Necessary methods for measuring the amount of postbiotics should be determined and developed. It should not be ignored that lipopolysaccharides present in Gram-negative bacteria can cause sepsis and toxic shock. Attention should be paid to product contamination. The effects of probiotics and postbiotics should be compared. It should be noted that different strains of the same bacteria can produce different postbiotics. A postbiotic can be a complex mixture, but what is responsible for the beneficial effects needs to be determined. The required dose should be determined. Bioavailability and bioaccessibility should be evaluated. How to evaluate the degree of microbial cell disruption after inactivation should be determined. Randomized controlled large-scale clinical studies should be conducted to demonstrate the clinical effects of postbiotics. It should be investigated whether existing postbiotic metabolites may enter the feedback loop and disrupt endogenous production [14, 69, 93–98].

## Conclusion

The microbiota uses dietary and gastrointestinal system factors in two different ways when dysbiosis is present and absent. Diet alters the microbiota and its metabolites. The link between diet, gut microbiota, and CRC has been established primarily as a relationship rather than a cause-effect relationship. It has been shown that various postbiotics can selectively induce apoptosis in CRC, prevent cell proliferation, growth, invasion, and migration, modulate the immune system, suppress carcinogenic signaling pathways, maintain intestinal epithelial integrity, and have a synergistic effect with chemotherapy drugs. However, it has been reported that some postbiotics are ineffective and may be risky regarding safety profile in some patients. Furthermore, there is insufficient information regarding the necessary dosage and whether it may induce a negative feedback effect in the body, inhibiting endogenous production, bioavailability, and bioaccessibility. Although numerous preclinical studies demonstrate the promising role of postbiotics in the prevention and treatment of CRC, clinical evidence remains scarce and is currently in its early stages. Therefore, there is a necessity for large-scale, randomized, double-blind clinical studies. Considering that not only bacteria but also mycobiota, virobiota, and archaeabiota contribute to both eubiosis and dysbiosis, postbiotic research based on these elements is anticipated to expand in the future.

## Data Availability

No datasets were generated or analyzed during the current study.

## Abbreviations

5-FU: 5-Fluorouracil
AKT: A serine/threonine protein kinase
AOM: Azoxymethane
APC: Adenomatous polyposis coli
BAD: BCL2-associated agonist of cell death
BAK: Bcl-2 homologous antagonist/killer
BAX: Bcl-2-associated X protein
Bcl-xl: B-cell lymphoma-extra-large
Bcl-2: B-cell lymphoma 2
CFS: Cell-free supernatant
CIMP: CpG island methylator phenotype
CIN: Chromosomal instability
CRC: Colorectal cancer
DCC: Deleted in colorectal cancer
DSS: Dextran sodium sulfate
EPS: Exopolysaccharides
GLUT-1: Glucose transporter-1
HDAC: Histone deacetylase
IL: Interleukin
MSI: Microsatellite instability
ISAPP: International Scientific Association for Probiotics and Prebiotics
SMAD2: Mothers Against Decapentaplegic homolog 2
K-ras: Kirsten rat sarcoma virus
MAPK: Mitogen-activated protein kinase
MyD88: Myeloid differentiation primary response protein 88
NADPH: Nicotinamide adenine dinucleotide phosphate
NF-κB: Nuclear factor kappa B
NLRP3: Nucleotide-binding-domain- and leucine-rich repeat-containing family, pyrin-domain-containing 3
OTUs: Operational taxonomic units
PARP: Poly ADP ribose polymerase
PI3K: Phosphatidylinositol 3-kinase
RAS: Rat sarcoma virus
ROS: Reactive oxygen species
SCFAs: Short-chain fatty acids
S-layers: Surface layers
SMO: Smoothened
TMAO: Trimethylamine-*N*-oxide
TLR-4: Toll-like receptor-4
TNF-α: Tumor necrosis factor alpha
Wnt: Wingless-related integration site
